# Antimicrobial-Loaded Polyacrylamide Hydrogels Supported on Titanium as Reservoir for Local Drug Delivery

**DOI:** 10.3390/pathogens12020202

**Published:** 2023-01-28

**Authors:** Irene E. Sille, Diego E. Pissinis, Natalia S. Fagali, Fiorela Ghilini, María Noel Urrutia, Patricia L. Schilardi

**Affiliations:** Instituto de Investigaciones Fisicoquímicas Teóricas y Aplicadas (INIFTA), CONICET-Facultad de Ciencias Exactas, Universidad Nacional de La Plata, Casilla de Correo 16, Sucursal 4, La Plata 1900, Argentina

**Keywords:** antibacterial, surface functionalization, hydrogel, silver nanoparticles, ampicillin

## Abstract

Arthroplasty is a highly successful treatment to restore the function of a joint. The contamination of the implant via bacterial adhesion is the first step toward the development of device-associated infections. The emerging concern about antimicrobial resistance resulted in a growing interest to develop alternative therapeutic strategies. Thus, the increment in the incidence of bacterial periprosthetic infections, the complexity of treating infections caused by organisms growing in biofilms, together with the rise in antibiotic resistant bacteria, expose the need to design novel surfaces that provide innovative solutions to these rising problems. The aim of this work is to develop a coating on titanium (Ti) suitable for inhibiting bacterial adhesion and proliferation, and hence, biofilm formation on the surface. We have successfully prepared polyacrylamide hydrogels containing the conventional antibiotic ampicillin (AMP), silver nanoparticles (AgNPs), and both, AMP and AgNPs. The release of the antibacterial agents from the gelled to aqueous media resulted in an excellent antibacterial action of the loaded hydrogels against sessile *S. aureus*. Moreover, a synergic effect was achieved with the incorporation of both AMP and AgNPs in the hydrogel, which highlights the importance of combining antimicrobial agents having different targets. The polyacrylamide hydrogel coating on the Ti surface was successfully achieved, as it was demonstrated by FTIR, contact angle, and AFM measurements. The modified Ti surfaces having the polyacrylamide hydrogel film containing AgNPs and AMP retained the highest antibacterial effect against *S. aureus* as it was found for the unsupported hydrogels. The modified surfaces exhibit an excellent cytocompatibility, since healthy, flattened MC3T3-E1 cells spread on the surfaces were observed. In addition, similar macrophage RAW 264.7 adhesion was found on all the surfaces, which could be related to a low macrophage activation. Our results indicate that AMP and AgNP-loaded polyacrylamide hydrogel films on Ti are a good alternative for designing efficient antibacterial surfaces having an excellent cytocompatibility without inducing an exacerbated immune response. The approach emerges as a superior alternative to the widely used direct adsorption of therapeutic agents on surfaces, since the antimicrobial-loaded hydrogel coatings open the possibility of modulating the concentration of the antimicrobial agents to enhance bacterial killing, and then, reducing the risk of infections in implantable materials.

## 1. Introduction

Arthroplasty is a highly successful surgical treatment to restore the function of a joint by resurfacing the bones or implanting a prosthesis. The benefits of this procedure include pain relief, the ability to carry out activities, and increased overall quality of life [1]. Prosthetic joint infection (PJI) is a serious condition for patients as well as the global healthcare industry. While a small minority of joint arthroplasties will become infected, the appropriate recognition and treatment of infections is critical to preserve or restore adequate function and prevent excessive morbidity [2,3]. Periprosthetic infection was estimated to represent 1% for hip arthroplasties and between 1% and 2% after knee arthroplasties. However, results from surveys among patients undergoing primary arthroplasty showed that infection rates may be higher. They accounted for 14.8% of revisions after hip arthroplasty and 25.2% after knee arthroplasty and are expected to increase due to the augmented prevalence of obesity, diabetes, and other comorbidities [4,5]. It is known that more than 65% of bacterial infections are caused by organisms growing in biofilms [6,7]. Biofilms are bacterial communities living immersed in an extracellular polymeric matrix that act as physical barriers to antibiotics treatment. The damage generated by biofilm-related infections in orthopedic practice is one of the most significant complications due to sequelae impacting joints and bones. The contamination of the implant via bacterial adhesion is the first step toward the development of PJI since the surfaces of commonly used orthopedic components such as titanium (Ti and its alloys), cobalt–chromium, stainless steel, are polymeric biomaterials (e.g., hydroxyapatite and polyethylene) are all susceptible to colonization by bacteria [8,9]. 

In order to eliminate or substantially reduce the bacterial attachment and biofilm formation on the implant surfaces, numerous investigations have focused on the fabrication of new antibacterial surfaces or on improving the performance of the existing ones. Some of the surface modifications mostly used are self-organized nanoarchitecture of Ti surfaces [10,11]; nanopatterned super hydrophobic surfaces [12]; nanopatterned smart polymer surfaces plus quaternary ammonium salt [13]; or polyzwitterionic coating [14], covalent modification of polyvinyl alcohol and cellulose on glass surfaces [15], plasma-based strategies [16], covalent bonding of broad-spectrum antimicrobials to Ti surfaces, such as SPI031 [17], amoxicillin [18], and vancomycin (VAN) [19]. The increment in antibiotic-resistant bacteria, particularly in hospital environments, results in a growing interest for the development of suitable prophylactic strategies to avoid this issue [20].

Silver nanoparticles (AgNPs) proved to be an interesting alternative antibacterial agent due to the low antimicrobial resistance expressed by bacteria. However, bacteria are able to adapt and even build up resistance to AgNPs. The possible bacterial resistance to AgNPs has been reported in recent years [21]. The mechanisms involved in the antimicrobial activity of AgNPs are complex and they are still under discussion. Some studies suggest that the nanoparticles interact directly with the cell wall, others attribute the antimicrobial action to the Ag(I) ions release from the nanoparticles, and others to the combined action of both effects [22,23]. AgNPs have been successfully used as an antibacterial agent in the treatment of water containing toxic heavy metal ions, organic fouling agents, and microbes [24], for moist wound healing [25], among other applications. In particular, several approaches have been explored to modify Ti surfaces with antimicrobial coatings containing AgNPs: the spontaneous adsorption of the nanoparticles on Ti/TiO_2_ surfaces [26], TiO_2_ nanotubular surface with VAN plus AgNPs [27], polymer coatings plus AgNPs [28], plasma electrolytic oxidation for Ca and Ag coatings [29], or Ti surface with AgNPs plus AMP [30]. In the search for new strategies to prevent infections, AgNPs have also been functionalized with antibiotics [31] and incorporated into hydrogels [32]. Although systemic pharmacological treatments (for instance, with AMP, gentamicin, or VAN) have been extensively used to prevent infections, strategies for the local administration of drugs have several advantages, including specific administration of one or more medicines at the site of the injury ensuring their penetration in the affected tissue, lower overall dosage requirements, and mitigation of potentially serious systemic side effects. In this regard, drug-loaded hydrogels have drawn considerable attention due to the possibility of modulating the local release of medicines through different mechanisms, according to the hydrogel nature. In order to achieve this goal, several types of hydrogels have been explored, such as degradable hydrogels [33], stimuli-responsive hydrogels [34], redox-responsive hydrogels [35], molecularly imprinted hydrogels [36], etc. In particular, the polyacrylamide hydrogel (PAAG) is especially interesting because it is a biocompatible [37], non-toxic, and non-degradable synthetic product [38]. It has been widely utilized in several biomedical applications, such as support for cellular and soft tissue growth [39], soft tissue filler in reconstructive and cosmetic surgery [40], and material for contact lenses [41], among others.

The increment in the incidence of bacterial PJI, the complexity of infection treatment when organisms growing in biofilms are involved, together with the increasing antibiotic-resistance of bacteria, lead to the design of novel surfaces able to provide innovative solutions to these emerging issues. The combination of different biocide agents having different antimicrobial mechanisms may be a useful strategy to address this growing problem.

In this work, we used a PAAG to design an antimicrobial coating on Ti suitable for inhibiting bacterial adhesion and proliferation, and hence, biofilm formation on the Ti surface. The coating consists of a thin layer of antimicrobial-loaded PAAG as reservoir for antibacterial agents. The hydrogel film contains AMP (conventional antibiotic) and/or AgNPs (alternative antimicrobial agent). Our approach involves the use of only PAAG without the need of polymer blends [33], grafting [42], etc., as is usual in most of the studies reported in the literature. It takes advantage of a simple and well-known methodology for the synthesis of PAAG in aqueous media, without the need for special techniques such as organic solvent evaporation through rotary evaporator [34], microwave assistance [42], UV irradiation [43], etc., which makes it appropriate for mass fabrication, since its intended potential application is the coating of Ti implant surfaces. In addition, the thin, non-degradable PAAG coating also overcomes the burst release found in degradable hydrogels [33], which could result in a harmful effect (possible cytotoxicity and inflammatory response).

## 2. Materials and Methods

### 2.1. Reagents

All solutions were prepared using ultrapure MilliQ^®^ water. All the following reagents were analytical grade and used as received, without further purification: silver nitrate (Sigma Aldrich), sodium citrate (J. T. Baker, Mexico), hydrogen peroxide 30% (Merck, Darmstadt, Germany), sodium borohydride (Sigma Aldrich, St. Louis, MO, USA), ampicillin (Laboratorios Fabra, Buenos Aires, Argentina), nutrient broth and nutrient agar (Laboratorios Britania, CABA, Argentina), acrylamide (Sigma Aldrich, St. Louis, MO, USA), *N,N*-methylene bis-acrylamide (Sigma Aldrich, St. Louis, MO, USA), *N,N,N,N*-tetra-methyl-ethylene-diamine (Merck, Darmstadt, Germany) and ammonium persulfate (Sigma Aldrich, Steinheim, Germany).

### 2.2. Silver Nanoparticles Preparation

AgNPs in aqueous solution were prepared as described elsewhere [28,30,44]. Briefly, the synthesis was carried out by adding the solutions in this order: 2.0 mL of 1.25 × 10^−2^ M sodium citrate, 5.0 mL of 3.75 × 10^−4^ M silver nitrate, and 5.0 mL of 5.0 × 10^−2^ M hydrogen peroxide. The silver reduction was achieved by adding 2.5 mL of freshly prepared 5.0 × 10^−3^ M sodium borohydride under vigorous magnetic stirring. After approximately 3 min, a stable color is reached, indicating the end of the synthesis. The colloidal dispersion was then dialyzed for 2 h to eliminate the excess reagents. The final Ag concentration in the nanoparticle dispersion was 14 ± 1 μg/mL.

### 2.3. Polyacrylamide Hydrogels Synthesis

**Figure 1:**
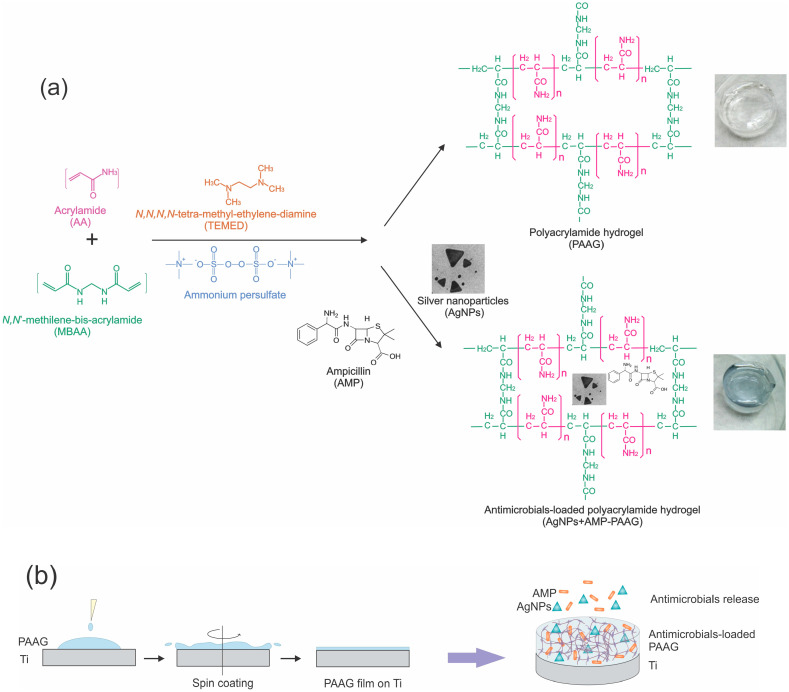
Schematic representation of (**a**) PAAG and antimicrobial-loaded PAAG preparation; (**b**) Ti coating and antimicrobials released from the AgNPs+AMP–PAAG–Ti.

The PAAG was obtained by the polymerization of acrylamide (AA) and *N,N*-methylene bis-acrylamide (MBAA, crosslinking agent) in aqueous phase following the methodology described by A. Lal Das et al. [45]. A total of 0.9 mL phosphate buffered (pH 7) aqueous solution containing AA (10% *w*/*v*) and MBAA (4% *w*/*v*) was stirred under N_2_ atmosphere and then mixed with 0.05 mL *N,N,N,N*-tetra-methyl-ethylene-diamine (TEMED, 1.25% *w*/*v*) (catalyst) and 0.05 mL ammonium persulfate (0.5% *w*/*v*) (initiator). The gelation time was about 2 min. The hydrogels were used as prepared without further purification. Once polymerization was finished, the samples were washed with ultrapure water. The general procedure is schematized in Figure 1a.

### 2.4. Antimicrobial-Loaded Polyacrylamide Hydrogel Preparation

#### 2.4.1. AMP-Loaded Polyacrylamide Hydrogel (AMP–PAAG)

The AMP–PAAG was prepared by the same procedure as described in Section 2.3, but adding AMP to the AA (10% *w*/*v*) and MBA (4% *w*/*v*) buffered solution to reach 2 mM AMP. Then, the catalyst and the initiator were added.

#### 2.4.2. AgNPs (AgNPs–PAAG)-Loaded Polyacrylamide Hydrogel

A buffered aqueous solution containing AA (10% *w*/*v*) and MBA (4% *w*/*v*) was added to a AgNPs pellet obtained from centrifugation of 1 mL of colloidal dispersion (14,000 rpm for 30 min). After that, the catalyst and the initiator were added (see Section 2.4).

#### 2.4.3. Polyacrylamide Hydrogel with AgNPs plus AMP (AgNPs+AMP–PAAG)

A buffered aqueous solution containing AA (10% *w*/*v*), MBA (4% *w*/*v*), and AMP (2 mM) were added to a AgNPs pellet obtained from centrifugation of 1 mL of colloidal dispersion (14,000 rpm for 30 min). After that, the catalyst and the initiator were added (see Section 2.3).

### 2.5. Antimicrobials Release

The release of AMP and silver was analyzed by immersing the AMP–PAAG and AgNPs–PAAG samples in appropriate aliquots (2 mL) of Milli-Q^®^ water for 1, 4, and 7 days. For 4 and 7 days, the water was renewed every 48 h: the supernatant liquid was removed and a new water aliquot was added. For each PAAG sample, all the collected aqueous supernatants were regrouped and then analyzed as described below. The results are expressed as released percentage equaling antimicrobial released amount/antimicrobial added amount ×100.

#### 2.5.1. Silver Quantification

Aqueous samples originated from AgNPs–PAAG water exposure were treated with H_2_O_2_ to oxidize all the released nanoparticles [30]. Each supernatant was acidified with 2% HNO_3_ and the amount of silver in the resulting solutions was measured. Total silver quantification was carried out by ICP-OES. Data are expressed as mean ± standard deviation.

#### 2.5.2. AMP Quantification

For the aim of monitoring the AMP release, a Prominence equipment from Shimadzu (Japan) (solvent delivery module LC-20AT, on-line degasser DGU-20A5, communications bus module CBM-20, autosampler SIL-20A HT, column oven CTO-10AS VP, and photodiode array (PDA) detector SPD-M20A) was employed. A Synergi Polar-RP column (ether-linked phenyl phase with polar endcapping, 150 × 4.6 mm, 4 μm, Phenomenex) was used for separation and quantification of AMP from the HPLC runs. The column temperature was set at 25 °C and the flow rate at 0.6 mL/min. A solution containing ammonium acetate (10 mM, pH = 4.0 ± 0.1)–acetonitrile 90-10 was used as mobile phase.

The in vitro release of AMP from AMP–PAAG was investigated by measuring the amount of the antibiotic released to Milli-Q^®^ using HPLC with UV detection at 256 nm. Two independent experiments were performed and samples were injected in duplicate. The data were expressed as the mean of all the values obtained. For calibration, aliquots having different volumes of a standard solution of AMP were injected. The calibration curve was obtained by plotting the area under each peak vs. the known amount of AMP. Data are expressed as mean ± standard deviation.

### 2.6. Hydrogel Swelling Measurements

The swelling assays were carried out in three different media: ultrapure water, phosphate-buffered saline solution (PBS), and simulated body fluid (SBF). The SBF solution was prepared according to Kokubo et al. [46]. PAAG samples (1 cm^3^) were dried until constant mass and accurately weighted. After that, each sample was immersed in each medium. The mass of each sample was verified every 60 min by eliminating the adsorbed liquid with soft tissue paper and weighting. The equilibrium swelling was reached when the mass of the swollen samples did not change after 3 measurements. The swelling was expressed as

$$Swelling\%=\frac{m_{s}-m_{0}}{m_{0}}\times 100$$

where *m_s_* is the mass of the swollen sample and *m*_0_ is the mass of the dried sample.

### 2.7. Titanium Surface Preparation

The substrates were Ti discs (Johnson-Mathey, London, UK, 99.7%) 1 cm in diameter and 0.25 mm in thickness. The substrates were first polished with abrasive paper, sonicated in ultrapure water for 15 min, and then polished at mirror grade with 1 μm diamond paste. After that, the substrates were sonicated in water and then in ethanol for 15 min and, finally, thoroughly rinsed and air-dried. 

### 2.8. Titanium Surfaces Coated with PAAG Film (Spin Coating Technique)

The clean Ti discs (see Section 2.6) were placed in a spin coater (Laurell WS-400B, North Wales, UK) and 75 µL of the solution containing AA, MBAA, catalyst, and redox initiator (see Section 2.3), which were poured on the substrates and left 1 min. Then, the substrates were spun from 0 rpm to 2000 rpm by using a 5 rpm/s speed ramp and subsequently kept at 2000 rpm for 5 min. After that, the Ti samples coated by the film were washed by pouring 75 µL of water on the hydrogel film and spun by using the same procedure as described above. The modified substrate was dried in the air at room temperature (Figure 1b). The same procedure described in Section 2.4 was carried out to prepare antimicrobial-loaded PAAG films. From now, the samples will be named PAAG–Ti for titanium covered by the PAAG film; AMP–PAAG–Ti for titanium covered by the AMP-loaded PAAG film; AgNPs–PAAG–Ti for titanium covered by the AgNP-loaded PAAG film; and AgNPs+AMP–PAAG–Ti for titanium covered by the AgNP and AMP-loaded PAAG film. 

### 2.9. Contact Angle Measurements

Contact angle measurements were carried out with a Ramé-Hart 2900 goniometer by dropping 2 μL of Milli-Q^®^ water on each substrate. Triplicate assays were performed.

### 2.10. Spectroscopic Measurements

UV–vis spectra of the AgNPs dispersions were acquired with a Perkin Elmer Lambda 35 Spectrophotometer. Fourier transform infrared (FTIR) spectra were obtained from a Varian 660 spectrometer equipped with an attenuated total reflection (ATR) accessory (MIRacle ATR, Pike Technologies) and with a ZnSe prism.

### 2.11. Atomic Force Microscopy (AFM) Imaging 

AFM imaging was carried out by using a Nanoscope V microscope (Bruker, MA, USA) operating in Tapping^®^ mode in air. Images were taken at 1 Hz with silicon tips (RTESP, 215–254 kHz and 20–80 N/m).

### 2.12. Bacterial Culture

*Staphylococcus aureus* (*S. aureus*, ATCC 25923) was grown in nutrient broth at 37 °C in a rotary shaker (200 rpm). Then, the bacterial suspension was adjusted to 10^8^ colony-forming units (CFU)/mL in a fresh growth medium and used immediately. 

#### 2.12.1. Kirby–Bauer Tests and Bacterial Quantification (Unsupported Hydrogels)

In a Petri dish with nutrient agar (Britania), 100 µL of inoculum (optical density, OD = 3) was spread with a sterile swab. PAAG or antimicrobial-loaded PAAG samples (1 cm^3^) were placed at the center of the Petri dish and incubated for 24 h at 37 °C. In order to quantify the viable bacteria adhered on the PAAG and antimicrobial-loaded PAAG, the samples were placed in 2.0 mL of nutrient broth/sterile water (1:1) inoculated with *S. aureus* (OD = 0.1) and incubated 24 h at 37 °C. Then, the number of adhered bacteria was determined through quantification by the serial dilution method and viable count. The experimental methodology has been detailed in previous works [47,48].

#### 2.12.2. Bacterial Attachment on Modified Titanium Surfaces

A drop of 100 μL of the bacterial suspension was seeded onto each modified Ti disk and left for 2 h at 37 °C to allow for bacterial adhesion. The substrates with attached bacteria were gently washed by immersion in double-distilled sterile water to remove the cells that were not irreversibly attached to the surface. 

##### Viable Sessile Bacteria

The number of viable sessile bacteria was determined through quantification by the serial dilution method and plate counting after the detachment of cells by sonication, according to the protocol previously developed in our laboratory [48]. Bare Ti disks were used as control. Three experiments were performed in independent trials. Data are expressed as mean ± standard deviation.

##### Live/Dead BacLight Bacterial Assay

Fluorescence imaging of *S. aureus* grown on substrates for 2 h was performed by using the FilmTracer LIVE/DEAD viability kit (Invitrogen). The staining solution was prepared as indicated in manufacturer protocol, by mixing 3 μL of component A (SYTO 9) and 3 μL of component B (propidium iodide) in 1 mL of double-distilled sterile water. After bacterial attachment, 40 μL of the staining mixture was gently poured on each substrate and then they were kept in the dark for 15 min at room temperature. After that, the biofilmed substrates were rinsed with sterile water. Fluorescent bacteria were visualized by epifluorescence with an Olympus BX-51 microscope. The microscope filters used were UMWG2 (excitation 510−550 nm and emission 590 nm) and U-MWB2 (excitation 460−490 and emission 520). Bacteria were kept hydrated throughout the entire procedure.

### 2.13. Cell Culture Assays

Mouse preosteoblast cell line MC3T3-E1 and macrophages RAW 264.7 were grown as monolayers in T-25 flasks with Dulbecco’s modified Eagle’s medium (DMEM) culture medium (GIBCO-BRL, Los Angeles, USA) supplemented with 10% inactivated fetal calf serum (Natocor, Villa Carlos Paz, Córdoba, Argentina), 50 IU/mL penicillin, and 50 μg/mL streptomycin sulfate, hereafter named as complete culture medium (CCM), in a humidified incubator at 37 °C and in a 5% CO_2_ atmosphere. Viable cells were counted in a Neubauer hemocytometer by the exclusion of Trypan Blue (Sigma-Aldrich, St. Louis, MO, USA) method.

For these analyses, 4 × 10^4^ cells/cm^2^ were cultured on Ti discs, with and without surface modifications, in 6-well culture plates. The cells were grown at 37 °C in a 5% CO_2_ humid atmosphere in CCM. After 24 h of incubation, the attached cells were stained with acridine orange (Sigma, St Louis, MO, USA) and immediately examined by epifluorescence microscopy (Olympus BX51, Olympus Corp., Tokyo, Japan) equipped with an appropriate filter, connected to an Olympus DP73 color video camera (Olympus Corp., Tokyo, Japan). Images were taken immediately after opening the microscope shutter and the number of viable attached cells was measured with Image J software. Three experiments were performed in independent trials. Data are expressed as mean ± standard error of the mean (SEM). Statistical differences were analyzed using multiple comparisons of Bonferroni with 99.9% of confidence. There are no statistically significant differences between data sharing identical letters in the graph.

## 3. Results and Discussion

### 3.1. Antibacterial Effect of Antimicrobial-Loaded PAAG

**Figure 2:**
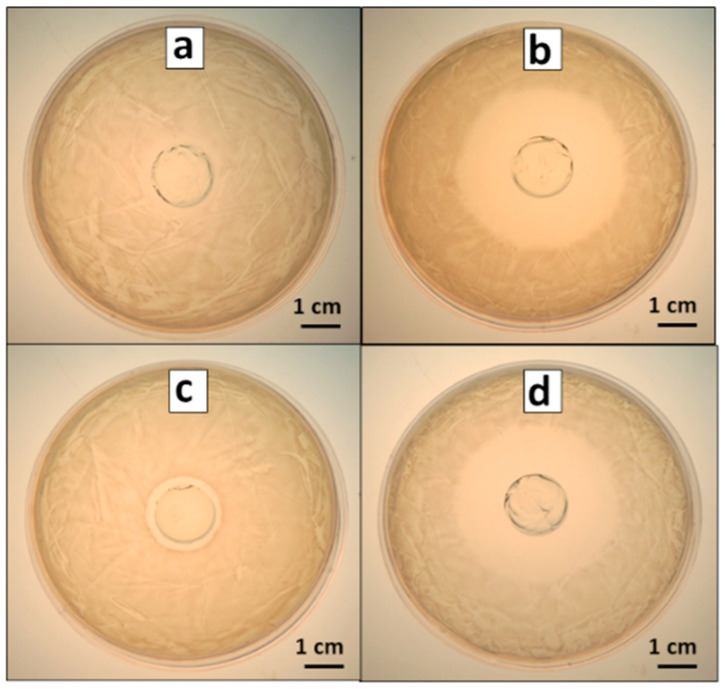
Inhibition halo in *S. aureus* culture on gelled media. (**a**) PAAG, (**b**) AMP–PAAG, (**c**) AgNPs–PAAG, (**d**) AgNPs+AMP–PAAG.

We analyzed the incorporation of the antimicrobials into hydrogel samples and then the ability of such materials to inhibit bacterial proliferation. To this end, qualitative assays were carried out through Kirby–Bauer tests in *S. aureus* cultures exposed to PAAG (control), AMP–PAAG, AgNPs–PAAG, and AgNPs+AMP–PAAG. Figure 2 shows that all the antimicrobial-loaded samples produced an inhibition zone around the samples, indicating that the antimicrobials were successfully incorporated to the hydrogels and are also able to be released from the samples. As expected, no inhibition zone was found for PAAG control samples (Figure 2a), while the highest antibacterial activity was found for AMP–PAAG (Figure 2b) and AgNPs–AMP–PAAG (Figure 2d). These results can be explained by taking into account that the diffusion rate in gelled media should be higher for the small AMP molecules than for the larger AgNPs [49]. On the other hand, the AgNPs’ antibacterial effect has been attributed to both the release of Ag(I) ions into the medium, which affects the bacterial viability by interaction with proteins [50], affecting the respiratory process [51] and/or interfering in DNA replication [52] as well as to the AgNPs themselves, through the internalization into the cell and/or the interaction with the bacterial cell wall [53]. In this regard, the smallest inhibition zone observed for AgNPs–PAAG can be attributed not only to a low diffusion of AgNPs in the hydrogel network but also to a low Ag(I) effective concentration since the agar employed for halo assays contains chloride ions, which may react with Ag(I) forming insoluble AgCl.

**Figure 3:**
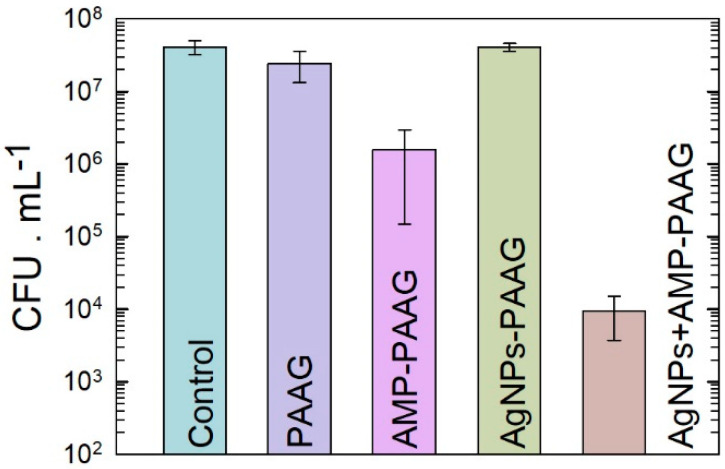
Quantification of viable *S. aureus* after 24 h of PAAG and antimicrobial-loaded PAAG incubation in liquid media. Data are expressed as mean ± standard deviation.

Additionally, we carried out a quantitative analysis of viable planktonic cells exposed to the PAAG and antimicrobial-loaded PAAG samples. The results are presented in Figure 2. As expected from the Kirby–Bauer assays, the PAAG has no significant antibacterial effect compared with the control (growth control). The PAAG and AgNPs–PAAG samples exhibited a similar number of CFU × mL^−1^ of viable bacteria, indicating that AgNPs–PAAG did not have a significant antibacterial effect on *S. aureus* cultures. On the contrary, the AMP–PAAG decreased 1.5 orders the number of viable bacteria compared with the control, indicating that AMP was able to be released into the culture medium. The highest antibacterial effect corresponds to AgNPs–AMP–PAAG, which exhibited a 4 orders decrease in the number of viable bacteria. Thus, according to Bonapace’s criteria, a synergistic effect is reached when AMP and AgNPs are combined since the bacterial viability decreased more than 2 log units with respect to the initial inoculum and the less effective treatment (AMP or AgNPs). (Figure 3).

### 3.2. PAAG Swelling and AMP and Ag Release from AMP–PAAG and AgNPs–PAAG

**Figure 4:**
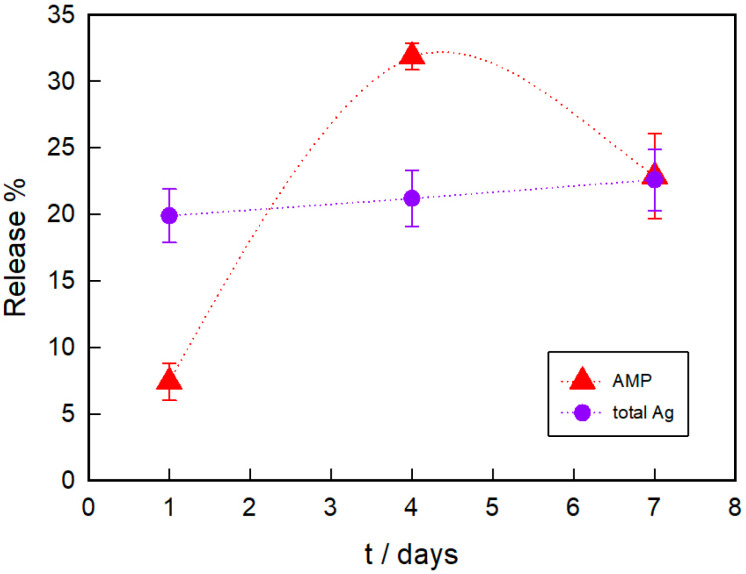
Release kinetics of AMP and AgNPs (as total Ag) from AMP–PAAG and AgNPs–PAAG, respectively. Data are expressed as mean ± standard deviation.

Figure 4 shows the released percentage of both AgNPs and AMP from the hydrogel. As it can be seen, the total Ag amount is held at about 20% as time increases, indicating that after 24 h no further release is produced. However, the amount of AMP rises from 7.4% to 31.8% as time goes from 1 to 4 days. After a week, only 22.9% AMP is found in the aqueous media, a value lower than that measured on the fourth day. Regarding this, the AMP stability in aqueous solution should be taken into account. Kang et al. measured the remaining amount of AMP dissolved in sterile water after 7 days at room temperature and found that 20% of the original amount of AMP breaks down [54]. Thus, some released AMP could be degraded after 7 days in the aqueous media. At this point, it is worth mentioning that a lower release of AMP compared with AgNPs leads to a higher antimicrobial activity, which can be interpreted in terms of the different bacterial susceptibility of *S. aureus* to these agents. 

**Figure 5:**
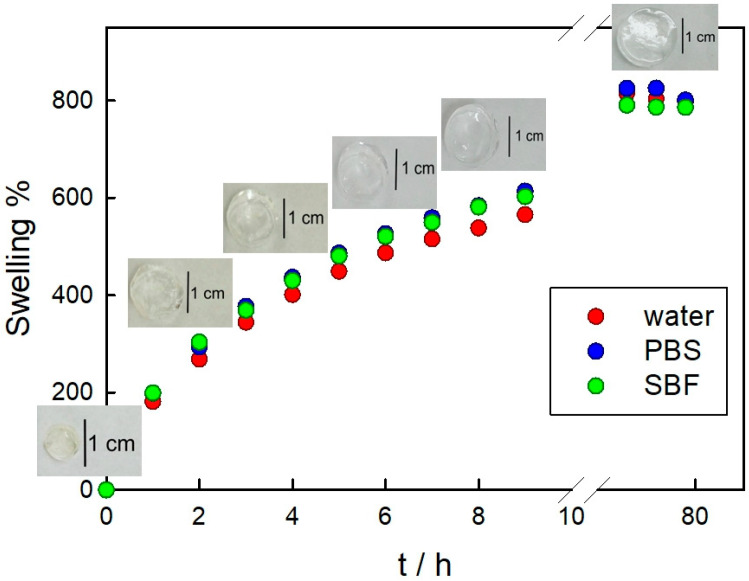
Swelling profile of PAAG in three media: water, PBS and SBF. Insets: images of PAAG swollen in water.

The release of entrapped drugs is directly related to the degree of swelling of the hydrogel. Swelling depends on the balance between forces restricting the deformation of the network and osmosis that results in water absorption. As the hydrogel swells, the mesh size increases, promoting diffusion-controlled delivery. Thus, we have analyzed the swelling behavior of PAAG in three relevant media for this work: ultrapure water, PBS, and SBF. The swelling profiles in these media are presented in Figure 5, where a similar trend can be observed for the three assayed conditions. However, at the first steps of hydration, the values at each time corresponding to PBS and SBF are slightly higher than those corresponding to water. In fact, Penkavova et al. reported that polyacrylamide hydrogels swell more in NaCl solutions than in water [55]. This behavior has been attributed to the formation of an electric double layer around polar groups of the polymer chain, such as C=O and C-N, which screens the polymer–polymer interactions [56]. The equilibrium swelling is reached after 73 h. Importantly, we have rehydrated PAAG and AgNPs+AMP–PAAG samples stored for more than two years and found similar results to those corresponding to the swelling in water of the freshly prepared hydrogels (data not shown).

### 3.3. Hydrogel Film on Titanium Physicochemical Characterization

The modification of Ti substrates with PAAG films or antimicrobial-loaded PAAG films was carried out by spin-coating. The approach comprises two simultaneous processes: radial liquid flow and solvent evaporation, resulting in a controlled thickness of the resulting film [57]. The technique is widely used to generate films on different substrates [58].

**Figure 6:**
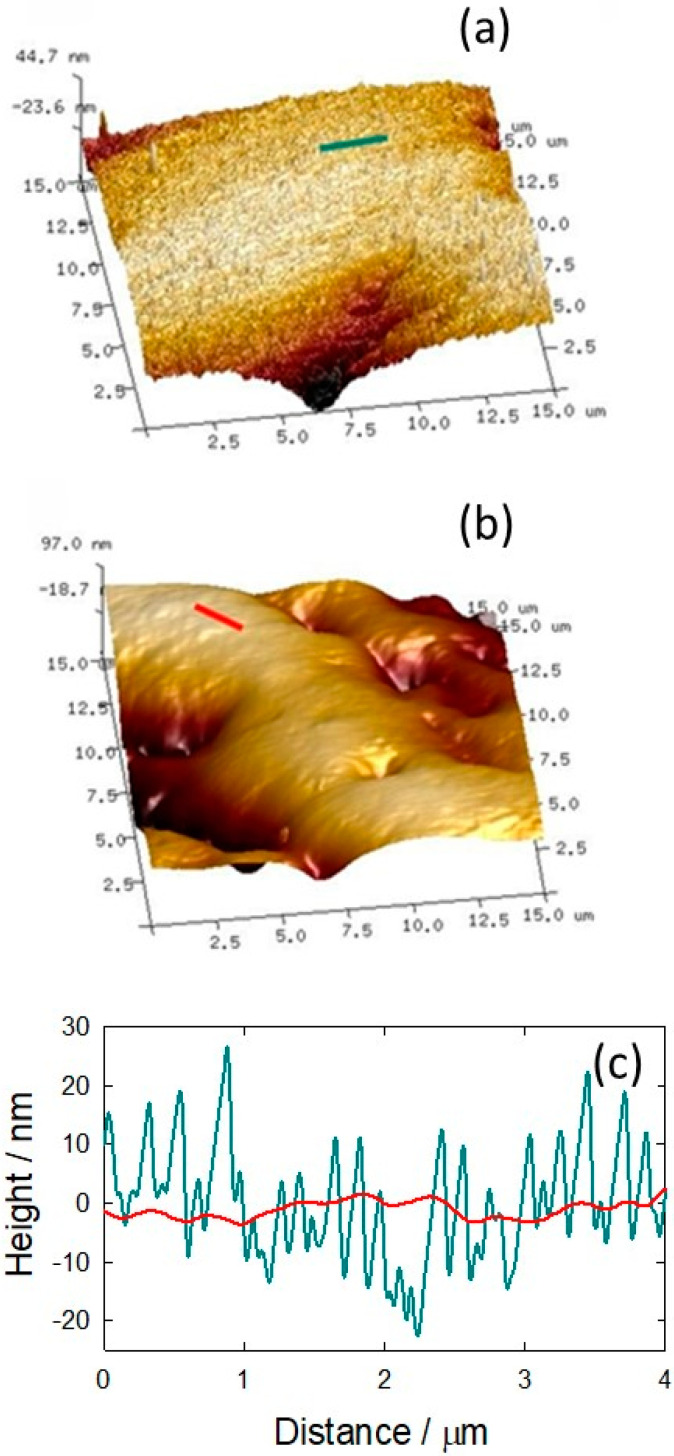
AFM topographic image (15 μm × 15 μm) of (**a**) Ti surface and (**b**) PAAG–Ti surface; (**c**) Cross-sectional analysis of Ti and PAAG–Ti surfaces along the cyan line in (**a**) and along the red line in (**b**), respectively.

In order to analyze the topography of a Ti surface before and after the PAAG coating, AFM measurements were carried out. AFM images (Figure 6) of bare Ti (Figure 6a) and PAAG-modified Ti (Figure 6b) exhibit a topographical change of the surface after coating. Ti exhibits a rather rough surface, mainly due to mechanical polishing, whereas in PAAG–Ti a smoother surface can be observed, which indicates the presence of a hydrogel film on the sample. This was confirmed by cross-sectional analysis, where the polishing features were lost in the modified substrates (Figure 6c). On the other hand, wetting properties depend on surface roughness and chemical composition. The modification of these surface parameters would lead to changes in the contact angle, and thus, in the wettability. The measurement of the wetting properties of bare and coated samples reveals a higher hydrophobicity for PAAG–Ti than Ti, since the contact angle increased from 50.74° to 72.64° after the substrate modification. The change in hydrophobicity could be explained by a decrease in the roughness of the Ti surface [59,60,61], and the presence of a higher percentage of hydrophobic groups on the surface due to dehydrated PAAG film [62]. 

**Figure 7:**
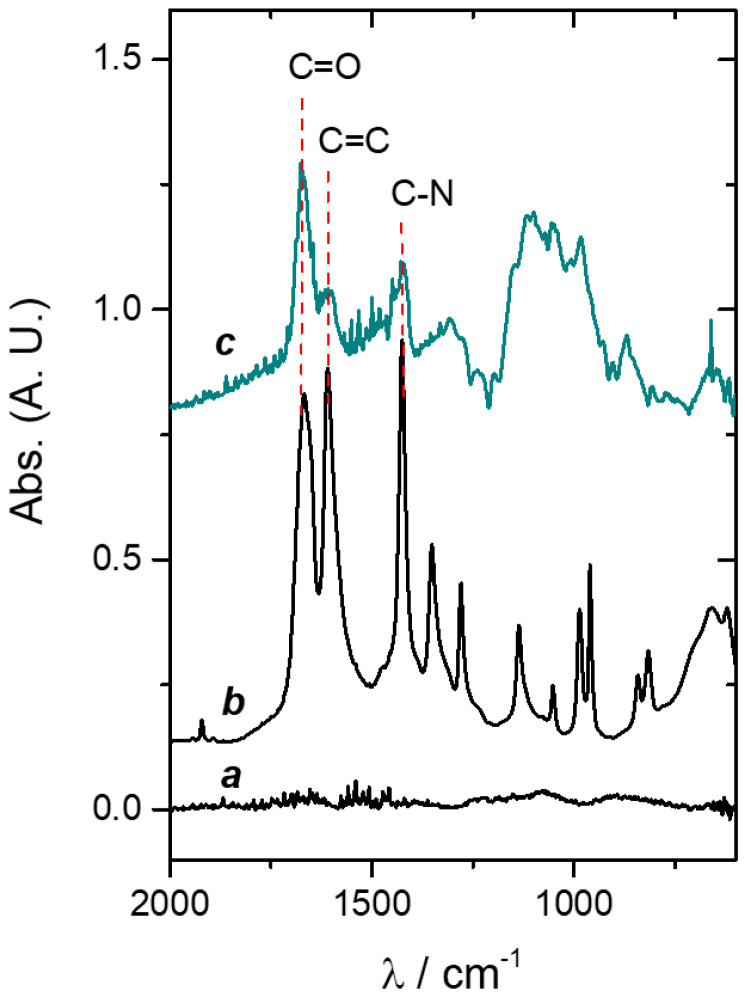
FTIR spectra of (**a**) Ti; (**b**) AA on Ti (drop casting); and (**c**) PAAG–Ti.

The evaluation of the chemical composition of the surface evidences the presence of the surface coating. Thus, FTIR–ATR measurements were carried out. Figure 7 shows the spectra corresponding to Ti, AA on Ti (drop casting), and PAAG–Ti. It can be observed that the characteristic bands due to PAAG appear in the 1400 and 1600 cm^−1^ region (Figure 7, line c) [63,64]. The typical bands at 1660 cm^−1^ correspond to the C=O stretching vibrations [65] and 1426 cm^−1^ is assigned to C-N stretching vibrations [65]. In the spectrum of AA deposited on Ti by drop casting (Figure 7, line b), the band at 1648 cm^−1^ corresponding to the C=C stretching vibrations is observed [64]. In the PAAG spectrum, this band diminished significantly, which would indicate the successful polymerization and the effective washing process to remove AA residues. 

### 3.4. Antibacterial Effect of Functionalized Surfaces

**Figure 8:**
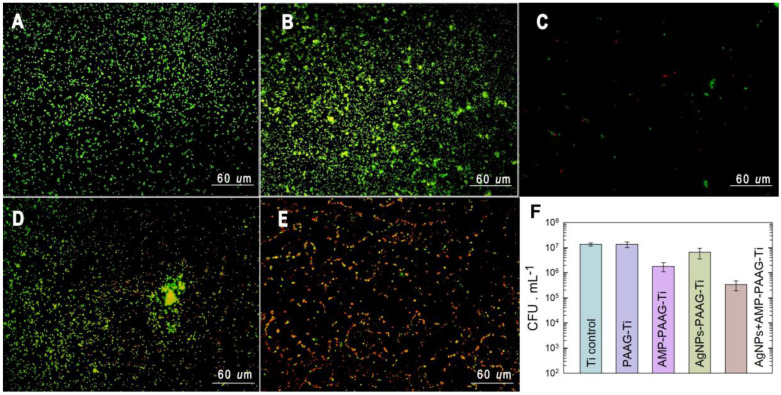
(**A**–**E**) Epifluorescence images of sessile bacteria after 2 h of incubation in *S. aureus* culture. (**A**) Ti; (**B**) PAAG–Ti; (**C**) AMP–PAAG–Ti; (**D**) AgNPs–PAAG–Ti; (**E**) AgNPs+AMP–PAAG–Ti. Green: live bacteria; red: dead bacteria. The images taken with each filter were superimposed. (**F**) Quantification of viable bacteria measured after 2 h in the *S. aureus* culture on each surface. Data are expressed as mean ± standard deviation.

Epifluorescence microscopy was carried out to qualitatively analyze dead bacteria in relation to the initially attached cells on the same substrate (Figure 8). As can be seen, *S. aureus* was able to attach and remain alive (green cells) on Ti (Figure 8A) and PAAG–Ti (Figure 8B). On AMP–PAAG–Ti (Figure 8C), a small number of sessile bacteria are observed, some of them non-viable (red cells). Importantly, on AgNPs–AMP–PAAG–Ti samples (Figure 8E), most of the attached bacteria are red, indicating an excellent antibacterial effect of the surface. 

The quantitative analysis of the performance of the modified Ti surfaces against *S. aureus* adhesion and proliferation was evaluated by plate counting (Figure 8F). The number of viable bacteria counted for PAAG–Ti did not present a significant difference with respect to the control (Ti). This result indicates that the PAAG has no antibacterial activity *per se*. The incorporation of AMP into the hydrogel produced a decrease in the viability of sessile bacteria of approximately one order when compared to the control. Conversely, the number of viable bacteria on AgNPs–PAAG–Ti does not significantly differ from those corresponding to Ti and PAAG–Ti samples. However, as expected from epifluorescence microscopy assays, AgNPs–AMP–PAAG–Ti samples behave as good antibacterial coating, evidenced by the decrease of 2 log in the number of viable bacteria in relation to the control. Similar results were previously found by our group for multifunctionalized Ti samples prepared through the direct adsorption of AMP and AgNPs on the surface [30]. The enhanced antimicrobial effect produced when both AMP and AgNPs are incorporated into the PAAG hydrogel highlights the importance of combining antimicrobial agents having different targets. AMP belongs to β-lactam antibiotics that disrupt the bacterial cell wall synthesis by covalent binding to penicillin-binding proteins (PBPs), enzymes involved in the last stages of peptidoglycan cross-linking in both, Gram-negative, and Gram-positive bacteria [66]. The β-lactams not only inhibit the PBPs, but also induce a toxic malfunctioning of the target biosynthetic machinery involving an ineffective cycle of cell wall synthesis, depleting cellular resources and then bolstering the antibiotic killing activity [67,68]. On the other hand, the mechanism of action of AgNPs is not fully understood yet, but the antimicrobial action is associated with four processes, namely (1) nanoparticle attachment on the surface of the cell wall and membrane; (2) AgNP internalization, producing the disruption of intracellular structures and biomolecules disruption; (3) reactive oxygen species and free radical production due to oxidative stress caused by AgNPs; (4) modulation of signal transduction pathways. Moreover, once these mechanisms have been triggered, the immune system of the host is stimulated, causing an inflammatory reaction, which further assists in the killing of the microorganisms [21].

### 3.5. Cytotoxicity Assays

#### 3.5.1. Pre-Osteoblast Adhesion

**Figure 9:**
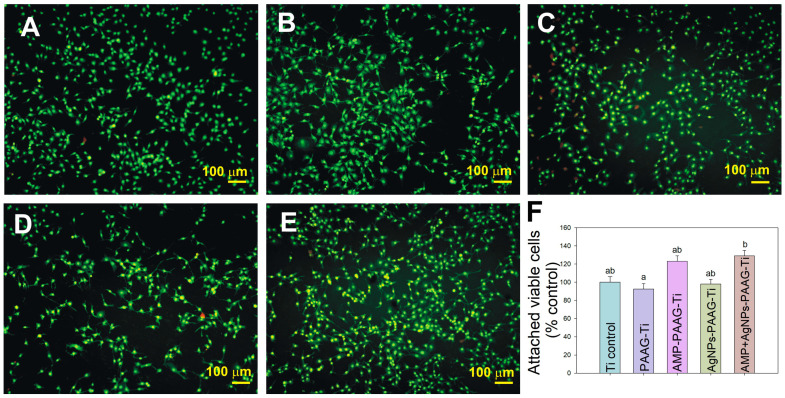
Epifluorescence images of pre-osteoblast cells (MC3T3-E1) cultured on the modified Ti surfaces: (**A**) Ti control (bare Ti); (**B**) PAAG–Ti; (**C**) AMP–PAAG–Ti; (**D**) AgNPs–PAAG–Ti; (**E**) AgNPs+AMP–PAAG–Ti. (**F**) Quantification of viable attached pre-osteoblast cells (MC3T3E-1) growing on modified Ti surfaces expressed as a percentage of Ti control. Data are expressed as mean ± standard error of the mean (SEM). Statistical differences were analyzed using multiple comparisons of Bonferroni with 99.9% of confidence. There are no statistically significant differences between data sharing identical letters in the graph.

It is commonly accepted that Ti and Ti alloys are biocompatible and not cytotoxic implantable materials [69,70]. Initial osteoblast adhesion on the Ti implant surface is determinant for long-term stability and other processes involved in osseointegration, including spreading, migration, proliferation, and differentiation. It is known that the ability of cells to adhere to surfaces is influenced by the surface features, which affect cell proliferation and differentiation capacity [71]. In order to determine the cytocompatibility of coatings on MC3T3-E1 pre-osteoblasts, cells were cultured on each modified Ti surface and, after incubation time, they were stained with acridine orange and observed with an epifluorescence microscope. Modified Ti surfaces were compared with bare Ti surfaces (Ti control). Microphotographs of representative samples are shown in Figure 9. The cell morphology in all the surfaces exhibited the characteristics of healthy cells [72], flattened and spread on the surfaces. 

The percentage of attached viable cells on the PAAG–Ti surface (92 ± 6%) was similar to that of the Ti control (100 ± 6%) (Figure 9F), without significant statistical differences. Thus, PAAG coating on a Ti surface exhibited a suitable cytocompatibility. The lack of cytotoxicity in PAAG–Ti samples is not surprising since polyacrylamide is widely used in cell biology to cultivate cells on soft surfaces [39] due to its biocompatibility [39]. Additionally, PAAG has been used in the biomedical area as an injectable implant, in drug treatment and delivery systems, in cell-based studies, ophthalmic surgery, wound dressing, etc. [73,74].

The addition of only AMP seems to enhance the cell attachment (123 ± 6%) although it has not exhibited a statistically significant difference. The presence of AgNPs in PAAG coating did not produce a significantly diminished cell viability (98 ± 5%) referred to Ti control. These results mean that AMP and AgNPs contained in PAAG coating do not have a significant cytotoxic effect on cultured pre-osteoblast cells. A significant increase in the number of attached cells on the AgNPs+AMP–PAAG–Ti surface was observed. On that surface, there was a rise in the number of attached pre-osteoblasts of about 37% over the PAAG–Ti sample, with a statistically significant difference. This result indicates that the simultaneous presence of AMP and AgNPs on PAAG coating is not significantly cytotoxic for pre-osteoblast cells and may help in the osseointegration process. Concerning this, it has been demonstrated that AgNPs are able to regulate the proliferation and differentiation of mesenchymal stem cells, leading to osteoinductive properties [75]. 

#### 3.5.2. Macrophage Adhesion

**Figure 10:**
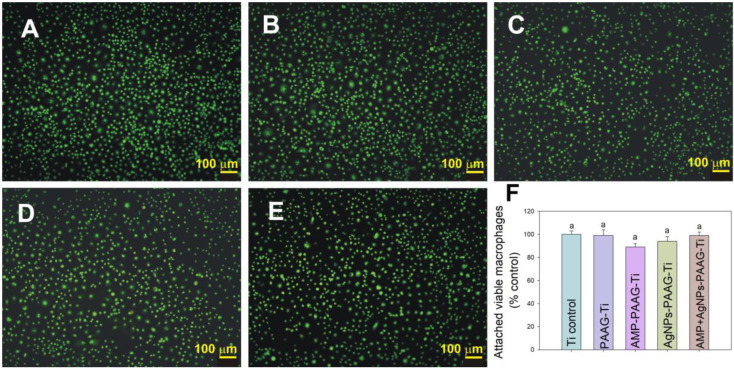
Epifluorescence images of RAW 264.7 macrophages cultured on the modified Ti surfaces: (**A**) Ti control; (**B**) PAAG–Ti; (**C**) AMP–PAAG–Ti; (**D**) AgNPs–PAAG–Ti; (**E**) AgNPs+AMP–PAAG–Ti. (**F**) Quantification of viable attached RAW 264.7 macrophage cells growing on modified Ti surfaces expressed as percentage of Ti control. Data are expressed as mean ± SEM. Statistical differences were analyzed using multiple comparisons of Bonferroni with 99.9% of confidence. There are not statistically significant differences between data sharing identical letters in the graph.

The immune system may recognize Ti implants as foreign bodies. Therefore, for non-degradable biomaterials, the interaction with immune cells as macrophages is an important aspect to take into account in the compatibility evaluation. Macrophages are central regulators of the immune response during infection and wound healing. Therefore, it is essential to choose biomaterials that stimulate the macrophage response as little as possible for long-term implantation [76]. A suitable response of macrophages and other cells such as neutrophils, lymphocytes, or monocytes is crucial to avoid encapsulation processes and to elicit a favorable osseointegration and healing response. Therefore, the effect of modified surfaces on RAW 164.7 macrophage adhesion was analyzed. Figure 10A–E show epifluorescence images of macrophages attached to the control and the modified samples. The number of attached macrophages on each sample did not present statistically significant differences between the samples (Figure 10F). This result indicates that the PAAG-modified surfaces with AMP and/or AgNPs would not induce increased macrophage recruitment compared with the Ti control, and hence, it would not produce an abnormal immune response. Additionally, in all cases, macrophages exhibited a low spreading morphology and they conserved their native spherical shape, which could be related to low macrophage activation.

## 4. Conclusions

We have successfully prepared polyacrylamide hydrogels containing AMP, AgNPs, and both AMP and AgNPs. The release of the antibacterial agents from the gelled to aqueous media resulted in an excellent antibacterial action of the loaded hydrogels against sessile *S. aureus*. Moreover, a synergic effect was achieved with the incorporation of both AMP and AgNPs in the hydrogel (AgNPs+AMP–PAAG), which highlights the importance of combining antimicrobial agents having different targets.

The polyacrylamide hydrogel coating on the Ti surface was successfully achieved, as it was demonstrated by FTIR, contact angle, and AFM measurements. The modified Ti surfaces having the polyacrylamide hydrogel film containing AgNPs and AMP retained the highest antibacterial effect against *S. aureus*, as it was found for the bulk hydrogels. The modified surfaces exhibit excellent cytocompatibility, since healthy, flattened cells spread on the surfaces were observed. In addition, similar macrophage adhesion was found on all the surfaces, where cells depicted a low spreading morphology and conserved their native spherical shape, which could be related to low macrophage activation. This result indicates that the PAAG-modified surfaces with AMP and/or AgNPs would not induce increased macrophage recruitment compared with the Ti control, and hence, it would not produce an abnormal immune response.

Our results indicate that AgNPs+AMP–PAAG films on Ti are a good alternative for designing efficient antibacterial surfaces. The direct adsorption of antimicrobials on the metal surfaces leads to limited surface concentration, and hence, limited effect on bacterial viability. The antimicrobial-loaded hydrogel coatings open the possibility of modulating the concentration of the antimicrobial agents to enhance bacterial killing. In this sense, it is worth stressing the importance of using non-degradable hydrogels, which would prevent the burst release possibly produced in degradable hydrogels, where the drug delivery is produced by both elution/diffusion and degradation. Thus, a slower release of the antimicrobial agents from no degradable hydrogels is expected, which in turn would lead to an action sustained over time.
